# The first coordination complex of (5*R*,6*R*,7*S*)-5-(furan-2-yl)-7-phenyl-4,5,6,7-tetra­hydro-[1,2,4]triazolo[1,5-*a*]pyrimidin-6-amine with zinc(II) acetate-chloride

**DOI:** 10.1107/S2056989021012226

**Published:** 2021-11-23

**Authors:** Mariia O. Shyshkina, Svitlana V Shishkina, Konstantin S. Ostras, Nikolay Yu. Gorobets, Valentyn A. Chebanov, Sergey M. Desenko

**Affiliations:** a SSI Institute for Single Crystals, NAS of Ukraine, 60 Nauky ave., Kharkiv 61001, Ukraine

**Keywords:** (5*R*,6*R*,7*S*)-5-(furan-2-yl)-7-phenyl-4,5,6,7-tetra­hydro-[1,2,4]triazolo[1,5-α]pyrimidin-6-amino­zinc(II)acetate-chloride, polymeric coordination complex, mol­ecular structure, crystal structure, Hirshfeld surface analysis

## Abstract

The title complex, systematic name *catena*-poly[[[acetato­chlorido­zinc(II)]-μ-(5*R*,6*R*,7*S*)-5-(furan-2-yl)-7-phenyl-4,5,6,7-tetra­hydro­[1,2,4]triazolo[1,5-*a*]py­rimi­din-6-amine] monohydrate], {[Zn(C_2_H_3_O_2_)Cl(C_15_H_15_N_5_O)]·H_2_O}_*n*_, is the first coordination complex in which the neutral tetra­hydro­triazolo­pyrimidine derivative acts as bridging ligand between two zinc mol­ecules. As a result, polymeric chains of the coordination complex are found. The coordination of the zinc metal atom occurs with the lone pairs of the triazolo nitro­gen atom and amino group. The positive charge of the zinc atom is compensated by the chlorine anion and deprotonated acetic acid. The coordination complex exists as a monohydrate in the crystalline phase. The water mol­ecules bind neighbouring polymeric chains by the formation of O—H⋯O, O—H⋯Cl and N—H⋯O hydrogen bonds.

## Abstract 2

The first coordination complex with (5*R*,6*R*,7*S*)-5-(furan-2-yl)-7-phenyl-4,5,6,7-tetra­hydro-[1,2,4]triazolo[1,5-α]pyrimidin-6-amino as a bridged ligand coordinating two zinc atoms has been synthesized and studied.

## Chemical context

Multicomponent reactions of 3-amino-1,2,4-triazole and carbonyl compounds have divergent selectivity, allowing the synthesis of alternative products from the same set of starting reagents (Sedash *et al.*, 2012 ▸). Such a phenomenon is used in diversity-oriented synthesis to increase the mol­ecular space of biologically active compounds. In previous research, we suggested a plausible reaction mechanism for the annulation of triazole with a tetra­hydro­pyrimidine ring occurring in reactions of 3-amino-1,2,4-triazole, aromatic aldehydes and ketocompounds (Gümüş *et al.*, 2017*a* ▸,*b* ▸). Generally, such reactions proceed *via* the inter­mediate formation of a Schiff base from the amino­azole and the aldehyde. One of the key stages of the mechanism is a nucleophilic attack of the electron-rich enol carbon atom onto the electron-deficit azo­methine carbon, with the formation of a C—C bond in the cyclization. If the suggested hypothesis is true, other reagents with a polar C=C bond similar to the C=C bond in enoles should possess similar reactivity. Using this analogy, we performed a multicomponent reaction between 3-amino-1,2,4-triazole, β-nitro­styrene and furfural. As expected, a derivative of tetra­hydro-[1,2,4]triazolo[1,5-*a*]pyrimidine **1** was obtained in high regio- and stereoselectivity. Further reduction of the nitro group in this compound unexpectedly resulted in formation of the zinc polycomplex **2**. A single crystal of this compound was characterized by X-ray diffraction. 
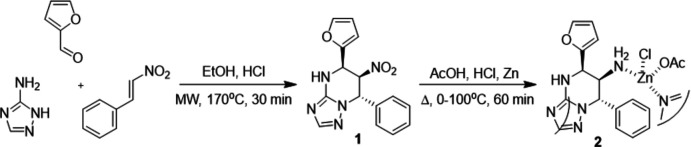


## Structural commentary

**Figure 1:**
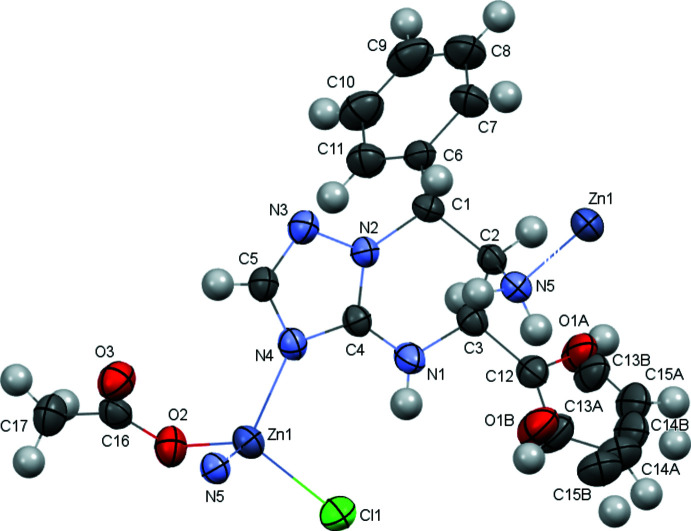
The mol­ecular structure of compound **2** (solvent mol­ecule and hydrogen atoms are omitted for clarity). Displacement ellipsoids are shown at the 50% probability level.

The title compound **2** is a coordination complex (Fig. 1 ▸) in which the zinc cation forms a salt with a chlorine anion and deprotonated acetic acid and is coordinated additionally by 5-furan-2-yl-7-phenyl-4,5,6,7-tetra­hydro-[1,2,4]triazolo[1,5-*a*]pyrimidin-6-­amine through inter­action with the electron lone pairs of the N4 atom of the triazole ring and the pyramidal amino group [the sum of bond angles, centered at the N5 atom, is 324°]. Thus, the zinc coordination polyhedron is tetra­hedral.

The tetra­hydro­pyrimidine ring of the neutral organic ligand adopts an asymmetric half-chair conformation (Fig. 1 ▸) with puckering parameters (Zefirov *et al.*, 1990 ▸) of *S* = 0.73, Θ = 35.0°, Ψ = 20.3°. The C2 and C1 atoms deviate from the mean-square plane of the remaining atoms of the ring by 0.76 and 0.18 Å, respectively. The three vicinal substituents have different orientations: the furan ring is located in the equatorial position, while the phenyl substituent and amino group are located in axial positions [the C4—N1—C3—C12_1/C12_2, N2—C1—C2—N5 and C4—N2—C1—C6 torsion angles are 161.4 (2), 161.4 (2), −78.2 (2) and 105.5 (3)° respectively].

The amino group and furan ring are *cis*-oriented. The furan ring is disordered over two positions with an occupancy ratio of 0.707 (11):0.293 (11) and twisted in relation to the N1—C3 endocyclic bond [the N1—C3—C12_1—C13_1 and N1—C3—C12_2—O1_2 torsion angles are −27.6 (9) and −36.5 (8)°, respectively]. This may be due to the strong bifurcated intra­molecular N—H⋯π hydrogen bonds (N5—H5*A*⋯C12_1/C12_2, N5—H5*B*⋯C13_1 and N*5*—H5*B*⋯O1_2; Table 1 ▸). The phenyl substituent is *trans*-oriented to the amino group and twisted with respect to the N2—C1 endocyclic bond [N2—C1—C6—C11 = −15.4 (4)°].

## Supra­molecular features

**Figure 2:**
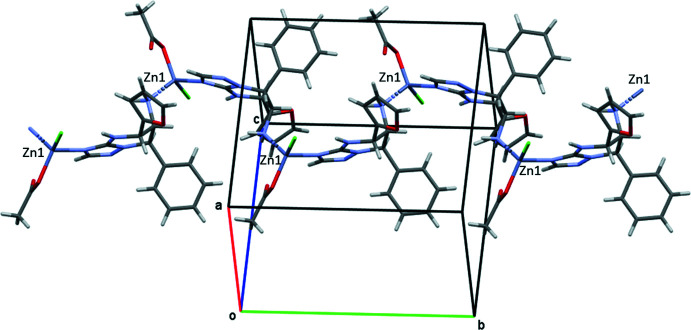
The chain of mol­ecules of **2** linked by N—H⋯Cl and N—H⋯O hydrogen bonds.

In the crystal, the coordination complex forms polymeric chains in the [010] direction, in which the neutral organic mol­ecule is bridged between two zinc cations (Fig. 2 ▸). The coordination polymer exists as a monohydrate in the crystal. The organic mol­ecule is linked to the chlorine and acetic anions by N1—H⋯Cl and N5—H5*A*⋯O3^i^ hydrogen bonds (Table 1 ▸). Neighbouring polymeric chains are connected through the water mol­ecules by O1*S*—H1*SA*⋯O2, O1*S*—H1*SB*⋯Cl and N5—H*5B*⋯O1*S* ^ii^ hydrogen bonds (Table 1 ▸).

## Hirshfeld surface analysis

**Figure 3:**
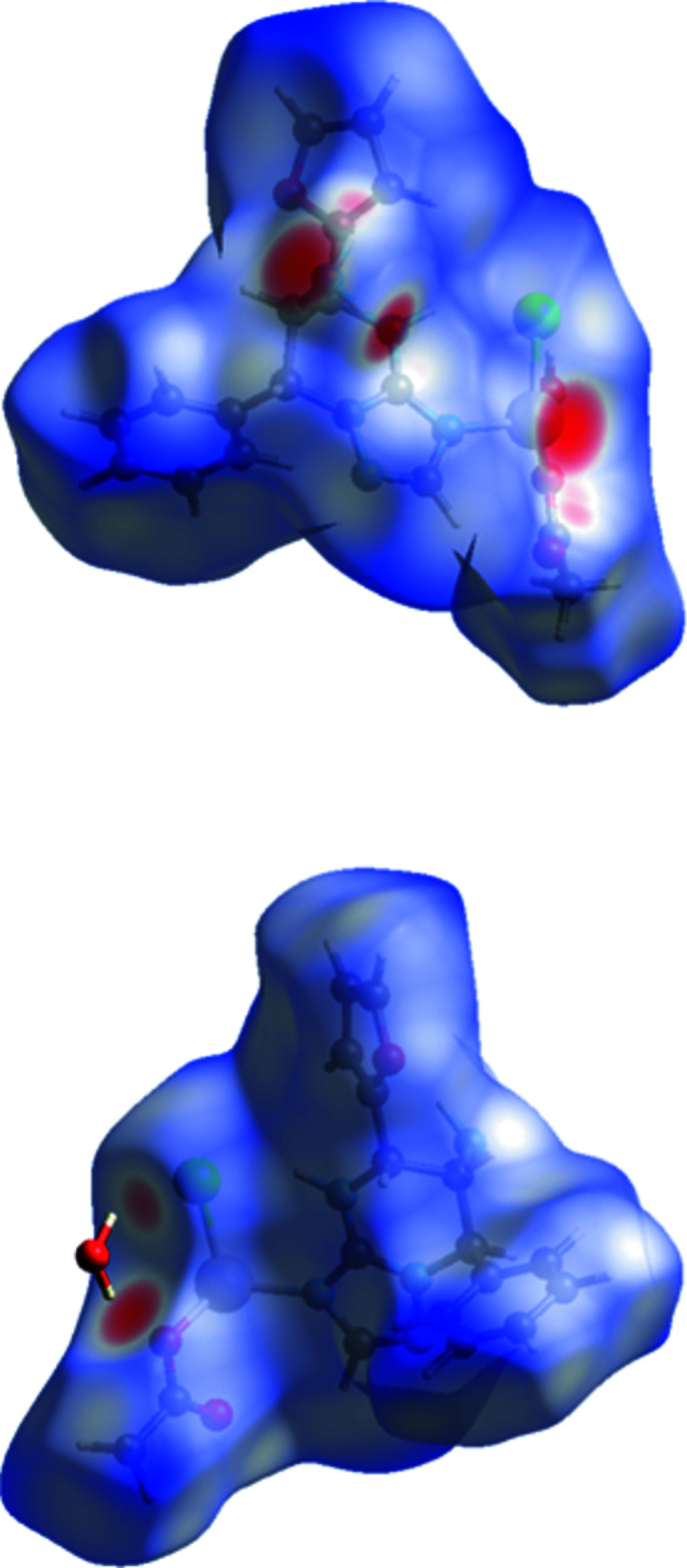
Two views of the Hirshfeld surface of compound **2** mapped over *d*_norm_ in the range −0.603 to 1.696 a.u.

Hirshfeld surface analysis (Turner *et al.*, 2017 ▸) was used to identify and visualize different types of intra- and inter­molecular inter­actions in the crystal structure. The mol­ecular Hirshfeld surface of the coordination complex was constructed using a standard surface resolution with three-dimensional *d*_norm_ surfaces. The areas coloured red on the *d*_norm_ surfaces correspond to strong inter­molecular O—H⋯O and N—H⋯O hydrogen bonds (Fig. 3 ▸). Bright red spots are also observed at the nitro­gen atom of the triazole ring, chlorine atom and one of the oxygen atoms of the acetic anion.

**Figure 4:**
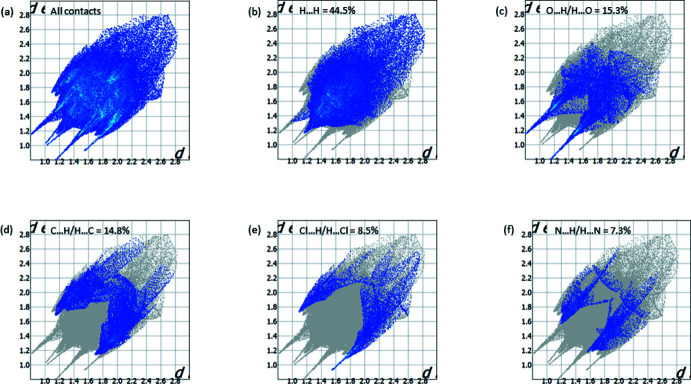
Two-dimensional fingerprint plots for compound **2** showing (*a*) all inter­actions, and delineated into (*b*) H⋯H, (*c*) O⋯H/H⋯O, (*d*) C⋯H/H⋯C, (*e*) Cl⋯H/H⋯Cl and (*f*) N⋯H/H⋯N contacts.

The pair of sharp spikes in the two-dimensional fingerprint plot (Fig. 4 ▸ *a*) indicates the presence of strong hydrogen bonds in the crystal structure. The main contribution to the Hirshfeld surface is provided by H⋯H contacts (44.5%), shown in Fig. 4 ▸ *b*. The contributions of O⋯H/H⋯O (15.3%) and C⋯H/H⋯C (14.8%) contacts associated with *X*—H⋯O and *X*—H⋯π hydrogen bonds are much smaller (Fig. 4 ▸ *c*, 4*d*). The smallest contributions in the total Hirshfeld surface are provided by Cl⋯H/H⋯Cl (8.5%) and N⋯H/H⋯N (7.3%) (Fig. 4 ▸ *e*, 4*f*) inter­actions associated with *X*—H⋯Cl and *X*—H⋯N hydrogen bonds.

## Database survey

A search of the Cambridge Structural Database (CSD Version 5.42, update of November 2020; Groom *et al.*, 2016 ▸) for the triazolo­pyrimidine fragment revealed 28 hits of which only 14 have a mol­ecular structure close to that of the neutral mol­ecules in the studied coordination complex [refcodes: CAGVIQ (Desenko *et al.*, 1999 ▸), EYATUU (Rudenko *et al.*, 2011 ▸), HEXKEA(Desenko *et al.*, 1994 ▸), HUVCAD (Gorobets *et al.*, 2010 ▸), OPIMIK (Lipson *et al.*, 2009 ▸), PUGDIF (Huang, 2009 ▸), QISRIW, QISRUI, QISSAP, QISSET (Zemlyanaya *et al.*, 2018 ▸), QOZMEY (Chen *et al.*, 2009 ▸), TOMPAN (Sakhno *et al.*, 2008 ▸), VEFXEL (Sedash *et al.*, 2012 ▸), YEHREK (Yu *et al.*, 2011 ▸)]. However, no triazolo­pyrimidine derivatives coord­inated to a metal atom have been deposited in the Cambridge Structural Database.

## Synthesis and crystallization

Microwave irradiation experiments were carried out using an Emrys^TM^ Creator EXP (Biotage, Uppsala) equipped with an outer IR temperature sensor. The reaction was performed in a sealed microwave process vial using the ‘very high’ mode, which decreased the initial power to 90 W. Reaction time under microwave conditions refers to the time that the reaction mixture was kept at the set temperature (fixed hold time).

**(5** ***R*** **,6** ***R*** **,7** ***S*** **)-5-(Furan-2-yl)-6-nitro-7-phenyl-4,5,6,7-tetra­hydro-[1,2,4]triazolo[1,5-*a*]pyrimidine (1):** In a microwave process vial, a volume of 0.2 mL of 40% HCl solution in EtOH was added to an equimolar mixture (4.0 mmol) of 3-amino-1,2,4-triazole, furfural, and β-nitro­styrene in 2.0 mL of methanol. The vessel was sealed and irradiated at 443 K for 40 min. After cooling, the precipitate that had formed was filtered off and washed with 2–3 mL of methanol. Drying gave compound **1** in a 41% yield, obtained in a mixture with its diastereomer in a ratio of 12:1. Pure compound **1** was obtained by recrystallization from ethanol.

**(5** ***R*** **,6** ***R*** **,7** ***S*** **)-5-(Furan-2-yl)-7-phenyl-4,5,6,7-tetra­hydro-[1,2,4]triazolo[1,5-** ***a*** **]pyrimidin-6-amine with zinc(II) acetate-chloride (2):** To a solution of 4.0 mmol of **1** in 5.0 mL of acetic acid was added 4.5 mL of concentrated hydro­chloric acid. The mixture was cooled down in an ice–water bath and 1.0 g of zinc dust was slowly added to the mixture portionwise. After the addition, the cooling bath was removed and the mixture was stirred for 30 min and then refluxed until the reducing agent was completely dissolved. The reaction mixture was left undisturbed overnight, and the single crystal used for the X-ray diffraction study was taken directly from the reaction mixture. The isolated yield of **2** was 67%.

## Refinement

Crystal data, data collection and structure refinement details are summarized in Table 2 ▸. All hydrogen atoms were located in difference-Fourier maps. They were included in calculated positions and treated as riding with C—H = 0.96 Å, *U*_iso_(H) = 1.5*U*_eq_(C) for methyl groups, O—H = 0.98 Å, *U*_iso_(H) = 1.5*U*_eq_(O) for the water mol­ecule, Car—H = 0.93 Å, C*sp* ^3^—H = 0.97 Å, N—H = 0.89 Å and *U*_iso_(H) = 1.2*U*eq(parent atom) for all other hydrogen atoms. The furan ring is disordered over two positions with an occupancy ratio of 0.707 (11):0.293 (11).

## Supplementary Material

Crystal structure: contains datablock(s) I. DOI: 10.1107/S2056989021012226/jy2010sup1.cif

Structure factors: contains datablock(s) I. DOI: 10.1107/S2056989021012226/jy2010Isup2.hkl

CCDC reference: 2122655

Additional supporting information:  crystallographic information; 3D view; checkCIF report

## Figures and Tables

**Table 1:** Hydrogen-bond geometry (Å, °)

*D*—H⋯*A*	*D*—H	H⋯*A*	*D*⋯*A*	*D*—H⋯*A*
O1*S*—H1*SA*⋯O2	0.98	1.91	2.830 (3)	155
O1*S*—H1*SB*⋯Cl1	0.98	2.47	3.321 (2)	144
N1—H1⋯Cl1	0.86	2.42	3.198 (2)	151
N5—H5*A*⋯O3^i^	0.89	2.48	2.961 (3)	114
N5—H5*A*⋯C12_1	0.89	2.49	2.966 (3)	114
N5—H5*A*⋯C13_1	0.89	2.67	3.422 (11)	143
N5—H5*A*⋯O1_2	0.89	2.33	3.086 (17)	144
N5—H5*A*⋯C12_2	0.89	2.49	2.966 (3)	114
N5—H5*B*⋯O1*S* ^ii^	0.89	2.03	2.904 (3)	169

Symmetry codes: (i) -x+1, y+{\script{1\over 2}}, -z+{\script{3\over 2}}; (ii) x, -y+{\script{1\over 2}}, z+{\script{1\over 2}}.

**Table 2:** Experimental details

Crystal data	
Chemical formula	[Zn(C_2_H_3_O_2_)Cl(C_15_H_15_N_5_O)]·H_2_O
*M*_r_	459.20
Crystal system, space group	Monoclinic, *P*2_1_/*c*
Temperature (K)	293
*a*, *b*, *c* (Å)	10.6267 (4), 12.8015 (5), 15.1646 (7)
β (°)	104.788 (4)
*V* (Å^3^)	1994.63 (15)
*Z*	4
Radiation type	Mo *K*α
μ (mm^−1^)	1.40
Crystal size (mm)	0.2 × 0.2 × 0.1

Data collection	
Diffractometer	Xcalibur, Sapphire3
Absorption correction	Multi-scan (*CrysAlis PRO*; Rigaku OD, 2018 ▸)
*T*_min_, *T*_max_	0.930, 1.000
No. of measured, independent and observed [*I* > 2σ(*I*)] reflections	15201, 4572, 3174
*R*_int_	0.042
(sin θ/λ)_max_ (Å^−1^)	0.649

Refinement	
*R*[*F* ^2^ > 2σ(*F* ^2^)], *wR*(*F* ^2^), *S*	0.042, 0.102, 1.02
No. of reflections	4572
No. of parameters	291
No. of restraints	90
H-atom treatment	H-atom parameters constrained
Δρ_max_, Δρ_min_ (e Å^−3^)	0.36, −0.32

Computer programs: *CrysAlis PRO* (Rigaku OD, 2018 ▸), *SHELXT2014/5* (Sheldrick, 2015*a* ▸), *SHELXL2017/1* (Sheldrick, 2015*b* ▸), *Mercury* (Macrae *et al.*, 2020 ▸) and *OLEX2* (Dolomanov *et al.*, 2009 ▸).

## supplementary crystallographic information

### Crystal data

**Table:**

[Zn(C_2_H_3_O_2_)Cl(C_15_H_15_N_5_O)]·H_2_O	*F*(000) = 944
*M**_r_* = 459.20	*D*_x_ = 1.529 Mg m^−^^3^
Monoclinic, *P*2_1_/*c*	Mo *K*α radiation, λ = 0.71073 Å
*a* = 10.6267 (4) Å	Cell parameters from 3540 reflections
*b* = 12.8015 (5) Å	θ = 3.1–29.5°
*c* = 15.1646 (7) Å	µ = 1.40 mm^−^^1^
β = 104.788 (4)°	*T* = 293 K
*V* = 1994.63 (15) Å^3^	Needle, colourless
*Z* = 4	0.2 × 0.2 × 0.1 mm

### Data collection

**Table:**

Xcalibur, Sapphire3 diffractometer	3174 reflections with *I* > 2σ(*I*)
Detector resolution: 16.1827 pixels mm^-1^	*R*_int_ = 0.042
ω scans	θ_max_ = 27.5°, θ_min_ = 3.0°
Absorption correction: multi-scan (CrysAlisPro; Rigaku OD, 2018)	*h* = −13→13
*T*_min_ = 0.930, *T*_max_ = 1.000	*k* = −16→16
15201 measured reflections	*l* = −19→19
4572 independent reflections	

### Refinement

**Table:**

Refinement on *F*^2^	90 restraints
Least-squares matrix: full	Hydrogen site location: mixed
*R*[*F*^2^ > 2σ(*F*^2^)] = 0.042	H-atom parameters constrained
*wR*(*F*^2^) = 0.102	*w* = 1/[σ^2^(*F*_o_^2^) + (0.0493*P*)^2^ + 0.1268*P*] where *P* = (*F*_o_^2^ + 2*F*_c_^2^)/3
*S* = 1.02	(Δ/σ)_max_ = 0.001
4572 reflections	Δρ_max_ = 0.36 e Å^−^^3^
291 parameters	Δρ_min_ = −0.32 e Å^−^^3^

### Special details

**Table:**

Geometry. All esds (except the esd in the dihedral angle between two l.s. planes) are estimated using the full covariance matrix. The cell esds are taken into account individually in the estimation of esds in distances, angles and torsion angles; correlations between esds in cell parameters are only used when they are defined by crystal symmetry. An approximate (isotropic) treatment of cell esds is used for estimating esds involving l.s. planes.

### Fractional atomic coordinates and isotropic or equivalent isotropic displacement parameters (Å^2^)

**Table:**

	*x*	*y*	*z*	*U*_iso_*/*U*_eq_	Occ. (<1)
Zn1	0.42716 (3)	0.16102 (3)	0.59755 (2)	0.03857 (12)	
Cl1	0.62402 (7)	0.20953 (6)	0.58000 (6)	0.0524 (2)	
O1S	0.4892 (2)	0.14368 (17)	0.36476 (14)	0.0552 (6)	
H1SA	0.416347	0.120957	0.389178	0.083*	
H1SB	0.562247	0.163167	0.416278	0.083*	
O2	0.32493 (19)	0.10659 (17)	0.48190 (13)	0.0481 (5)	
O3	0.18743 (19)	0.07023 (18)	0.56429 (15)	0.0545 (6)	
N1	0.5043 (2)	0.42425 (18)	0.62954 (16)	0.0437 (6)	
H1	0.560504	0.381973	0.617396	0.052*	
N2	0.3175 (2)	0.44947 (17)	0.68186 (15)	0.0342 (5)	
N3	0.2161 (2)	0.39097 (19)	0.69900 (16)	0.0424 (6)	
N4	0.3523 (2)	0.28948 (17)	0.64306 (15)	0.0358 (5)	
N5	0.5586 (2)	0.55569 (17)	0.79892 (14)	0.0352 (5)	
H5A	0.638491	0.541918	0.793789	0.042*	
H5B	0.526184	0.496510	0.814732	0.042*	
C1	0.3340 (2)	0.5612 (2)	0.70008 (18)	0.0350 (6)	
H1A	0.316410	0.574588	0.759441	0.042*	
C2	0.4782 (2)	0.5873 (2)	0.70857 (17)	0.0340 (6)	
H2	0.485507	0.663307	0.703691	0.041*	
C3	0.5248 (3)	0.5371 (2)	0.62890 (19)	0.0365 (6)	
H3	0.472603	0.565499	0.571084	0.044*	
C4	0.3965 (2)	0.3877 (2)	0.64934 (17)	0.0328 (6)	
C5	0.2433 (3)	0.2978 (2)	0.67508 (19)	0.0403 (6)	
H5	0.191826	0.240239	0.679448	0.048*	
C6	0.2415 (3)	0.6275 (2)	0.6302 (2)	0.0389 (6)	
C7	0.2274 (3)	0.7316 (2)	0.6495 (2)	0.0541 (8)	
H7	0.273434	0.758626	0.705375	0.065*	
C8	0.1466 (3)	0.7960 (3)	0.5875 (3)	0.0693 (11)	
H8	0.139303	0.866309	0.600944	0.083*	
C9	0.0773 (3)	0.7563 (3)	0.5065 (3)	0.0681 (10)	
H9	0.020910	0.799302	0.465039	0.082*	
C10	0.0898 (3)	0.6544 (3)	0.4856 (3)	0.0641 (10)	
H10	0.042548	0.628125	0.429733	0.077*	
C11	0.1729 (3)	0.5892 (3)	0.5472 (2)	0.0510 (8)	
H11	0.182172	0.519689	0.532211	0.061*	
O1_1	0.6872 (5)	0.6493 (4)	0.5947 (4)	0.0578 (14)	0.707 (11)
C12_1	0.6648 (3)	0.5593 (2)	0.63613 (19)	0.0404 (6)	0.707 (11)
C13_1	0.7773 (9)	0.5100 (9)	0.6723 (8)	0.064 (2)	0.707 (11)
H13_1	0.786583	0.444450	0.699558	0.076*	0.707 (11)
C14_1	0.8815 (6)	0.5769 (7)	0.6613 (6)	0.067 (2)	0.707 (11)
H14_1	0.970497	0.565320	0.682355	0.081*	0.707 (11)
C15_1	0.8216 (7)	0.6599 (5)	0.6139 (5)	0.0609 (17)	0.707 (11)
H15_1	0.864388	0.716553	0.596380	0.073*	0.707 (11)
O1_2	0.7572 (13)	0.4952 (13)	0.6906 (12)	0.054 (3)	0.293 (11)
C12_2	0.6648 (3)	0.5593 (2)	0.63613 (19)	0.0404 (6)	0.293 (11)
C13_2	0.7307 (17)	0.6428 (15)	0.6144 (16)	0.055 (3)	0.293 (11)
H13_2	0.693906	0.703607	0.585317	0.066*	0.293 (11)
C14_2	0.8683 (14)	0.6197 (14)	0.6447 (13)	0.055 (3)	0.293 (11)
H14_2	0.935927	0.660277	0.634767	0.065*	0.293 (11)
C15_2	0.8800 (12)	0.5283 (14)	0.6901 (10)	0.055 (3)	0.293 (11)
H15_2	0.957438	0.493801	0.716452	0.066*	0.293 (11)
C16_2	0.2172 (3)	0.0672 (2)	0.4915 (2)	0.0410 (7)	
C17_2	0.1314 (3)	0.0175 (3)	0.4078 (2)	0.0573 (8)	
H17A_2	0.124694	0.062911	0.356446	0.086*	
H17B_2	0.046435	0.005959	0.417039	0.086*	
H17C_2	0.168324	−0.048027	0.396537	0.086*	

### Atomic displacement parameters (Å^2^)

**Table:**

	*U*^11^	*U*^22^	*U*^33^	*U*^12^	*U*^13^	*U*^23^
Zn1	0.0440 (2)	0.03285 (19)	0.0390 (2)	−0.00017 (14)	0.01079 (14)	0.00118 (14)
Cl1	0.0451 (4)	0.0508 (5)	0.0657 (5)	−0.0013 (3)	0.0220 (4)	−0.0036 (4)
O1S	0.0621 (14)	0.0589 (15)	0.0466 (13)	−0.0016 (11)	0.0171 (11)	−0.0110 (10)
O2	0.0505 (12)	0.0500 (13)	0.0450 (12)	−0.0136 (10)	0.0144 (10)	−0.0036 (10)
O3	0.0477 (12)	0.0654 (15)	0.0517 (13)	−0.0037 (11)	0.0148 (10)	−0.0044 (11)
N1	0.0453 (13)	0.0337 (13)	0.0596 (16)	−0.0047 (11)	0.0275 (12)	−0.0088 (12)
N2	0.0324 (11)	0.0294 (12)	0.0413 (13)	−0.0018 (9)	0.0105 (10)	0.0006 (10)
N3	0.0356 (12)	0.0390 (14)	0.0541 (15)	−0.0041 (11)	0.0143 (11)	0.0018 (12)
N4	0.0386 (12)	0.0295 (12)	0.0387 (13)	−0.0032 (10)	0.0088 (10)	−0.0003 (10)
N5	0.0370 (12)	0.0307 (12)	0.0380 (13)	0.0005 (10)	0.0100 (10)	−0.0021 (10)
C1	0.0357 (14)	0.0313 (14)	0.0393 (15)	0.0032 (11)	0.0119 (12)	−0.0044 (12)
C2	0.0374 (14)	0.0258 (13)	0.0375 (15)	−0.0002 (11)	0.0075 (11)	0.0006 (11)
C3	0.0415 (15)	0.0324 (14)	0.0366 (15)	−0.0018 (12)	0.0121 (12)	0.0001 (12)
C4	0.0368 (14)	0.0303 (14)	0.0311 (14)	−0.0015 (11)	0.0081 (11)	0.0007 (11)
C5	0.0369 (14)	0.0369 (15)	0.0475 (17)	−0.0056 (12)	0.0118 (13)	0.0003 (13)
C6	0.0320 (14)	0.0336 (15)	0.0517 (18)	0.0024 (12)	0.0119 (13)	0.0030 (13)
C7	0.0497 (18)	0.0392 (18)	0.070 (2)	0.0094 (15)	0.0087 (16)	−0.0005 (16)
C8	0.055 (2)	0.043 (2)	0.108 (3)	0.0121 (17)	0.018 (2)	0.009 (2)
C9	0.0477 (19)	0.069 (3)	0.087 (3)	0.0123 (19)	0.015 (2)	0.028 (2)
C10	0.0494 (19)	0.073 (3)	0.064 (2)	0.0101 (18)	0.0042 (17)	0.013 (2)
C11	0.0472 (17)	0.0461 (18)	0.055 (2)	0.0086 (15)	0.0051 (15)	0.0027 (16)
O1_1	0.046 (2)	0.047 (2)	0.082 (3)	−0.011 (2)	0.019 (2)	0.016 (2)
C12_1	0.0424 (14)	0.0383 (15)	0.0449 (16)	−0.0020 (12)	0.0188 (13)	0.0012 (12)
C13_1	0.054 (4)	0.065 (4)	0.078 (5)	0.005 (3)	0.026 (3)	0.019 (3)
C14_1	0.055 (3)	0.065 (5)	0.085 (5)	0.003 (3)	0.023 (3)	0.008 (4)
C15_1	0.045 (3)	0.053 (3)	0.090 (4)	−0.014 (3)	0.028 (3)	0.006 (3)
O1_2	0.038 (5)	0.066 (5)	0.060 (6)	0.002 (4)	0.016 (4)	0.022 (4)
C12_2	0.0424 (14)	0.0383 (15)	0.0449 (16)	−0.0020 (12)	0.0188 (13)	0.0012 (12)
C13_2	0.044 (6)	0.048 (6)	0.064 (6)	0.000 (5)	−0.003 (6)	0.014 (5)
C14_2	0.041 (5)	0.061 (8)	0.061 (7)	0.000 (5)	0.010 (5)	0.019 (6)
C15_2	0.034 (5)	0.069 (7)	0.061 (6)	0.004 (5)	0.009 (5)	0.017 (5)
C16_2	0.0451 (16)	0.0306 (15)	0.0456 (18)	0.0043 (12)	0.0082 (14)	0.0021 (13)
C17_2	0.0482 (17)	0.059 (2)	0.060 (2)	−0.0102 (16)	0.0043 (15)	−0.0079 (17)

### Geometric parameters (Å, º)

**Table:**

Zn1—Cl1	2.2621 (8)	C6—C11	1.374 (4)
Zn1—O2	1.9413 (19)	C7—H7	0.9300
Zn1—N4	2.023 (2)	C7—C8	1.374 (5)
Zn1—N5^i^	2.046 (2)	C8—H8	0.9300
O1S—H1SA	0.9832	C8—C9	1.360 (5)
O1S—H1SB	0.9829	C9—H9	0.9300
O2—C16_2	1.293 (3)	C9—C10	1.357 (5)
O3—C16_2	1.224 (3)	C10—H10	0.9300
N1—H1	0.8600	C10—C11	1.388 (4)
N1—C3	1.461 (3)	C11—H11	0.9300
N1—C4	1.340 (3)	O1_1—C12_1	1.362 (5)
N2—N3	1.390 (3)	O1_1—C15_1	1.389 (6)
N2—C1	1.459 (3)	C12_1—C13_1	1.338 (10)
N2—C4	1.336 (3)	C13_1—H13_1	0.9300
N3—C5	1.301 (4)	C13_1—C14_1	1.442 (10)
N4—C4	1.337 (3)	C14_1—H14_1	0.9300
N4—C5	1.370 (3)	C14_1—C15_1	1.348 (8)
N5—H5A	0.8900	C15_1—H15_1	0.9300
N5—H5B	0.8900	O1_2—C12_2	1.380 (13)
N5—C2	1.474 (3)	O1_2—C15_2	1.374 (14)
C1—H1A	0.9800	C12_2—C13_2	1.364 (18)
C1—C2	1.542 (3)	C13_2—H13_2	0.9300
C1—C6	1.509 (4)	C13_2—C14_2	1.447 (15)
C2—H2	0.9800	C14_2—H14_2	0.9300
C2—C3	1.557 (4)	C14_2—C15_2	1.346 (15)
C3—H3	0.9800	C15_2—H15_2	0.9300
C3—C12_1	1.491 (4)	C16_2—C17_2	1.501 (4)
C3—C12_2	1.491 (4)	C17_2—H17A_2	0.9600
C5—H5	0.9300	C17_2—H17B_2	0.9600
C6—C7	1.381 (4)	C17_2—H17C_2	0.9600
			
O2—Zn1—Cl1	108.26 (6)	C11—C6—C7	118.5 (3)
O2—Zn1—N4	114.95 (9)	C6—C7—H7	119.4
O2—Zn1—N5^i^	111.81 (9)	C8—C7—C6	121.1 (3)
N4—Zn1—Cl1	105.83 (6)	C8—C7—H7	119.4
N4—Zn1—N5^i^	103.45 (9)	C7—C8—H8	120.2
N5^i^—Zn1—Cl1	112.44 (6)	C9—C8—C7	119.6 (4)
H1SA—O1S—H1SB	108.3	C9—C8—H8	120.2
C16_2—O2—Zn1	110.22 (17)	C8—C9—H9	119.7
C3—N1—H1	120.5	C10—C9—C8	120.5 (3)
C4—N1—H1	120.5	C10—C9—H9	119.7
C4—N1—C3	119.0 (2)	C9—C10—H10	119.9
N3—N2—C1	123.6 (2)	C9—C10—C11	120.3 (4)
C4—N2—N3	109.9 (2)	C11—C10—H10	119.9
C4—N2—C1	126.5 (2)	C6—C11—C10	120.0 (3)
C5—N3—N2	101.8 (2)	C6—C11—H11	120.0
C4—N4—Zn1	128.83 (17)	C10—C11—H11	120.0
C4—N4—C5	102.4 (2)	C12_1—O1_1—C15_1	106.1 (4)
C5—N4—Zn1	128.72 (19)	O1_1—C12_1—C3	114.6 (3)
Zn1^ii^—N5—H5A	108.2	C13_1—C12_1—C3	135.3 (5)
Zn1^ii^—N5—H5B	108.2	C13_1—C12_1—O1_1	110.1 (4)
H5A—N5—H5B	107.3	C12_1—C13_1—H13_1	126.1
C2—N5—Zn1^ii^	116.37 (16)	C12_1—C13_1—C14_1	107.7 (7)
C2—N5—H5A	108.2	C14_1—C13_1—H13_1	126.1
C2—N5—H5B	108.2	C13_1—C14_1—H14_1	127.5
N2—C1—H1A	107.7	C15_1—C14_1—C13_1	104.9 (6)
N2—C1—C2	107.3 (2)	C15_1—C14_1—H14_1	127.5
N2—C1—C6	113.1 (2)	O1_1—C15_1—H15_1	124.6
C2—C1—H1A	107.7	C14_1—C15_1—O1_1	110.8 (5)
C6—C1—H1A	107.7	C14_1—C15_1—H15_1	124.6
C6—C1—C2	113.1 (2)	C15_2—O1_2—C12_2	110.2 (9)
N5—C2—C1	110.2 (2)	O1_2—C12_2—C3	118.3 (6)
N5—C2—H2	107.7	C13_2—C12_2—C3	134.1 (8)
N5—C2—C3	112.6 (2)	C13_2—C12_2—O1_2	106.3 (8)
C1—C2—H2	107.7	C12_2—C13_2—H13_2	126.2
C1—C2—C3	110.6 (2)	C12_2—C13_2—C14_2	107.6 (11)
C3—C2—H2	107.7	C14_2—C13_2—H13_2	126.2
N1—C3—C2	109.0 (2)	C13_2—C14_2—H14_2	126.4
N1—C3—H3	108.8	C15_2—C14_2—C13_2	107.1 (12)
N1—C3—C12_1	109.6 (2)	C15_2—C14_2—H14_2	126.4
N1—C3—C12_2	109.6 (2)	O1_2—C15_2—H15_2	126.1
C2—C3—H3	108.8	C14_2—C15_2—O1_2	107.9 (11)
C12_1—C3—C2	111.9 (2)	C14_2—C15_2—H15_2	126.1
C12_1—C3—H3	108.8	O2—C16_2—C17_2	115.7 (3)
C12_2—C3—C2	111.9 (2)	O3—C16_2—O2	122.0 (3)
N2—C4—N1	121.9 (2)	O3—C16_2—C17_2	122.3 (3)
N2—C4—N4	109.9 (2)	C16_2—C17_2—H17A_2	109.5
N4—C4—N1	128.1 (2)	C16_2—C17_2—H17B_2	109.5
N3—C5—N4	116.0 (2)	C16_2—C17_2—H17C_2	109.5
N3—C5—H5	122.0	H17A_2—C17_2—H17B_2	109.5
N4—C5—H5	122.0	H17A_2—C17_2—H17C_2	109.5
C7—C6—C1	118.7 (3)	H17B_2—C17_2—H17C_2	109.5
C11—C6—C1	122.8 (3)		
			
Zn1—O2—C16_2—O3	2.8 (4)	C2—C3—C12_2—O1_2	84.5 (10)
Zn1—O2—C16_2—C17_2	−177.2 (2)	C2—C3—C12_2—C13_2	−80.4 (15)
Zn1—N4—C4—N1	−2.3 (4)	C3—N1—C4—N2	−10.4 (4)
Zn1—N4—C4—N2	179.18 (17)	C3—N1—C4—N4	171.3 (3)
Zn1—N4—C5—N3	−179.08 (19)	C3—C12_1—C13_1—C14_1	−176.4 (5)
Zn1^ii^—N5—C2—C1	−85.3 (2)	C3—C12_2—C13_2—C14_2	175.5 (10)
Zn1^ii^—N5—C2—C3	150.70 (17)	C4—N1—C3—C2	38.6 (3)
N1—C3—C12_1—O1_1	150.2 (4)	C4—N1—C3—C12_1	161.4 (2)
N1—C3—C12_1—C13_1	−27.6 (9)	C4—N1—C3—C12_2	161.4 (2)
N1—C3—C12_2—O1_2	−36.5 (10)	C4—N2—N3—C5	0.1 (3)
N1—C3—C12_2—C13_2	158.6 (15)	C4—N2—C1—C2	−19.9 (3)
N2—N3—C5—N4	−0.6 (3)	C4—N2—C1—C6	105.5 (3)
N2—C1—C2—N5	−78.2 (2)	C4—N4—C5—N3	0.8 (3)
N2—C1—C2—C3	47.0 (3)	C5—N4—C4—N1	177.8 (3)
N2—C1—C6—C7	166.3 (2)	C5—N4—C4—N2	−0.7 (3)
N2—C1—C6—C11	−15.4 (4)	C6—C1—C2—N5	156.3 (2)
N3—N2—C1—C2	158.2 (2)	C6—C1—C2—C3	−78.5 (3)
N3—N2—C1—C6	−76.3 (3)	C6—C7—C8—C9	1.2 (5)
N3—N2—C4—N1	−178.1 (2)	C7—C6—C11—C10	−1.3 (4)
N3—N2—C4—N4	0.4 (3)	C7—C8—C9—C10	−1.6 (5)
N5—C2—C3—N1	66.3 (3)	C8—C9—C10—C11	0.5 (6)
N5—C2—C3—C12_1	−55.1 (3)	C9—C10—C11—C6	0.9 (5)
N5—C2—C3—C12_2	−55.1 (3)	C11—C6—C7—C8	0.2 (5)
C1—N2—N3—C5	−178.3 (2)	O1_1—C12_1—C13_1—C14_1	5.6 (11)
C1—N2—C4—N1	0.2 (4)	C12_1—O1_1—C15_1—C14_1	3.2 (7)
C1—N2—C4—N4	178.8 (2)	C12_1—C13_1—C14_1—C15_1	−3.5 (11)
C1—C2—C3—N1	−57.5 (3)	C13_1—C14_1—C15_1—O1_1	0.1 (9)
C1—C2—C3—C12_1	−178.9 (2)	C15_1—O1_1—C12_1—C3	176.1 (4)
C1—C2—C3—C12_2	−178.9 (2)	C15_1—O1_1—C12_1—C13_1	−5.5 (8)
C1—C6—C7—C8	178.6 (3)	O1_2—C12_2—C13_2—C14_2	9 (2)
C1—C6—C11—C10	−179.6 (3)	C12_2—O1_2—C15_2—C14_2	7 (2)
C2—C1—C6—C7	−71.5 (3)	C12_2—C13_2—C14_2—C15_2	−5 (2)
C2—C1—C6—C11	106.9 (3)	C13_2—C14_2—C15_2—O1_2	−1 (2)
C2—C3—C12_1—O1_1	−88.7 (4)	C15_2—O1_2—C12_2—C3	−179.0 (10)
C2—C3—C12_1—C13_1	93.4 (9)	C15_2—O1_2—C12_2—C13_2	−10 (2)

Symmetry codes: (i) −*x*+1, *y*−1/2, −*z*+3/2; (ii) −*x*+1, *y*+1/2, −*z*+3/2.

### Hydrogen-bond geometry (Å, º)

**Table:**

*D*—H···*A*	*D*—H	H···*A*	*D*···*A*	*D*—H···*A*
O1*S*—H1*SA*···O2	0.98	1.91	2.830 (3)	155
O1*S*—H1*SB*···Cl1	0.98	2.47	3.321 (2)	144
N1—H1···Cl1	0.86	2.42	3.198 (2)	151
N5—H5*A*···O3^ii^	0.89	2.48	2.961 (3)	114
N5—H5*A*···C12_1	0.89	2.49	2.966 (3)	114
N5—H5*A*···C13_1	0.89	2.67	3.422 (11)	143
N5—H5*A*···O1_2	0.89	2.33	3.086 (17)	144
N5—H5*A*···C12_2	0.89	2.49	2.966 (3)	114
N5—H5*B*···O1*S*^iii^	0.89	2.03	2.904 (3)	169

Symmetry codes: (ii) −*x*+1, *y*+1/2, −*z*+3/2; (iii) *x*, −*y*+1/2, *z*+1/2.

## References

[bb1] Chen, Q., Jiang, L.-L., Chen, C.-N. & Yang, G.-F. (2009). *J. Heterocycl. Chem.* **46**, 139–148.

[bb2] Desenko, S. M., Lipson, V. V., Shishkin, O. V., Komykhov, S. A., Orlov, V. D., Lakin, E. E., Kuznetsov, V. P. & Meier, H. (1999). *J. Heterocycl. Chem.* **36**, 205–208.

[bb3] Desenko, S. M., Shishkin, O. V., Orlov, V. D., Lipson, V. V., Linderman, S. V. & Struchkov, Yu. T. (1994). *Khim. Get. Soedin., SSSR*, **7**, 981–986.

[bb4] Dolomanov, O. V., Bourhis, L. J., Gildea, R. J., Howard, J. A. K. & Puschmann, H. (2009). *J. Appl. Cryst.* **42**, 339–341.

[bb5] Gorobets, N. Yu., Sedash, Y. V., Ostras, K. S., Zaremba, O. V., Shishkina, S. V., Baumer, V. N., Shishkin, O. V., Kovalenko, S. M., Desenko, S. M. & Van der Eycken, E. V. (2010). *Tetrahedron Lett.* **51**, 2095–2098.

[bb6] Groom, C. R., Bruno, I. J., Lightfoot, M. P. & Ward, S. C. (2016). *Acta Cryst.* B**72**, 171–179. 10.1107/S2052520616003954 PMC4822653 27048719

[bb7] Gümüş, M. K., Gorobets, N. Y., Sedash, Y. V., Shishkina, S. V. & Desenko, S. M. (2017*b*). *Tetrahedron Lett.* **58**, 3446–3448.

[bb8] Gümüş, M. K., Gorobets, N. Y., Sedash, Y. V., Chebanov, V. A. & Desenko, S. M. (2017*a*). *Chem. Heterocycl. Compd*, **53**, 1261–1267.

[bb9] Huang, S. (2009). *Acta Cryst.* E**65**, o2671. 10.1107/S1600536809039373 PMC2971018 21578280

[bb10] Lipson, V. V., Karnozhitskaya, T. M., Shishkina, S. V., Shishkin, O. V. & Turov, A. V. (2009). *Izv. Akad. Nauk SSSR, Ser. Khim.* **58**, 1400–1404.

[bb11] Macrae, C. F., Sovago, I., Cottrell, S. J., Galek, P. T. A., McCabe, P., Pidcock, E., Platings, M., Shields, G. P., Stevens, J. S., Towler, M. & Wood, P. A. (2020). *J. Appl. Cryst.* **53**, 226–235. 10.1107/S1600576719014092 PMC6998782 32047413

[bb12] Rigaku OD (2018). *CrysAlis PRO.* Rigaku Oxford Diffraction, Yarnton, England.

[bb13] Rudenko, R. V., Komykhov, S. A., Musatov, V. I., Konovalova, I. S., Shishkin, O. V. & Desenko, S. M. (2011). *J. Heterocycl. Chem.* **48**, 888–895.

[bb14] Sakhno, Y. I., Desenko, S. M., Shishkina, S. V., Shishkin, O. V., Sysoyev, D. O., Groth, U., Kappe, C. O. & Chebanov, V. A. (2008). *Tetrahedron*, **64**, 11041–11049.

[bb15] Sedash, Y. V., Gorobets, N. Y., Chebanov, V. A., Konovalova, I. S., Shishkin, O. V. & Desenko, S. M. (2012). *RSC Adv.* **2**, 6719–6728.

[bb16] Sheldrick, G. M. (2015*a*). *Acta Cryst.* A**71**, 3–8.

[bb17] Sheldrick, G. M. (2015*b*). *Acta Cryst.* A**71**, 3–8.

[bb18] Turner, M. J., McKinnon, J. J., Wolff, S. K., Grimwood, D. J., Spackman, P. R., Jayatilaka, D. & Spackman, M. A. (2017). *CrystalExplorer17.* University of Western Australia. http://Hirshfeldsurface.net

[bb19] Yu, W., Goddard, C., Clearfield, E., Mills, C., Xiao, T., Guo, H., Morrey, J. D., Motter, N. E., Zhao, K., Block, T. M., Cuconati, A. & Xu, X. (2011). *J. Med. Chem.* **54**, 5660–5670. 10.1021/jm200696v PMC3158247 21786803

[bb20] Zefirov, N. S., Palyulin, V. A. & Dashevskaya, E. E. (1990). *J. Phys. Org. Chem.* **3**, 147–158.

[bb21] Zemlyanaya, N. I., Karnozhitskaya, T. M., Musatov, V. I., Konovalova, I. S., Shishkina, S. V. & Lipson, V. V. (2018). *Zh. Org. Khim.* **54**, 1241–1249.

